# Are YouTube Videos a Reliable Source for Obtaining Information About Colorectal Cancer Screening?

**DOI:** 10.7759/cureus.21550

**Published:** 2022-01-24

**Authors:** Omer Yalkin, Mustafa Y Uzunoglu, Nidal İflazoglu, Ebru Esen

**Affiliations:** 1 Surgical Oncology, Bursa City Hospital, Bursa, TUR; 2 General Surgery, Bursa City Hospital, Bursa, TUR; 3 Surgical Oncology, Gülhane Training and Research Hospital, Ankara, TUR

**Keywords:** quality, cancer, early detection of cancer, colorectal neoplasms, youtube

## Abstract

Objective: This study aimed to assess the content, quality, and reliability of YouTube videos on colorectal cancer screening. Colorectal cancer is the third most common cause of death worldwide.

Methods: A basic search was conducted on the YouTube^TM^ website on November 19, 2020, using the English keywords "colorectal cancer screening," without using any filter. The videos were categorized into five groups according to the source and then each video was evaluated by three physicians. The videos were compared in terms of the quality of the information, and their reliability and comprehensiveness, according to the sources.

Results: Of the 137 videos included in the study, 74 (54%) were categorized in the useful information group and 63 (46%) in the misleading information group. It was found that most (47.3%) of the videos in the useful information group were uploaded by academic-based sources. Conversely, most (46%) of the videos in the misleading information group were uploaded by for-profit companies, private hospitals, and medical advertisements. An analysis of the video features according to the upload source revealed that the total views (p=0.023), likes (p=0.004), and viewer engagement with the video (p=0.026) were higher in the public spotlight videos group.

Conclusionsː The quality of public spotlight videos with high viewing rates and audience interaction should be increased to inform the public. In addition, there is a need for videos containing comprehensive and accurate information to be uploaded to YouTube, which is an important source of information today, by universities, health organizations, and doctors who do not make a profit (financially) from the results.

## Introduction

Colorectal cancer (CRC) is the third most common cancer for both men and women and ranks third among cancer-related deaths. Every year there are approximately 900,000 CRC-related deaths globally [1]. According to 2020 Surveillance, Epidemiology, and End Results (SEER) data, CRC accounts for 8.2% of all cancer cases and 8.8% of cancer-related deaths. The five-year relative survival is 64.6% [2].

The mortality rate of CRC (the number of deaths per 100,000 people a year) has been declining for decades in both men and women. The most important reason for this decline is that screening methods have been developed and are being widely used to reach the whole community. By means of screening methods, colorectal polyps can be detected before they turn into cancer or at an early enough stage whereby existing cancers can be fully cured. Although the overall mortality rate continues to decline, CRC-related mortality rates in people under the age of 55 years have purportedly increased by 1% each year from 2008 to 2017 [3]. For this reason, changes were made to the CRC screening program by the American Cancer Society in 2018, reducing the starting age for screening for people at normal risk from 50 to 45 years [4]. CRC screening is an important preventive measure, recommended by the US Preventive Services Task Force [5]. The American College of Gastroenterology has recommended colonoscopy as the preferred screening method for CRC [6].

Despite these recommendations, CRC screening rates in general and the frequency of colonoscopy screening, in particular, are below optimal [7]. The types of tests used for CRC screening are fecal-based tests (detecting hemoglobin in blood coming from any lesion or DNA changes suggesting malignancy) and visual contact tests. Visual contact tests are procedures performed using an endoscope (direct visualization with a small camera that visualizes lesions in the colon and also allows biopsy/lesion removal during testing) and radiological imaging procedures to detect lesions (virtual imaging) [8]. The inadequate rate of CRC screening due to lack of social awareness presents a difficulty for some screening methods (e.g., preparation for colon cleansing is required before a colonoscopy) [9].

Today, the internet is almost the first point of reference for learning about health problems. YouTube offers an obvious advantage in this regard. As of the beginning of 2021, the total number of views of the top 10 most-watched YouTube health channels had exceeded 1.9 billion [10].YouTube users are known to account for 95% of all internet users [11]. Studies have reported that patients use YouTube videos as a source of information for their health problems, in order to seek solutions before seeing a doctor, share experiences, and even buy treatment [12,13]. This study aimed to assess the content, quality, and reliability of YouTube videos on colorectal cancer screening.

## Materials and methods

A basic search was conducted on the YouTube website on November 19, 2020, using the English keywords "colorectal cancer screening," without using any filter. Anticipating that a normal YouTube user would not use any filters when searching for videos on CRC screening, the first 200 videos obtained by searching with no filter were saved as a playlist to be examined by the physicians participating in the study. The videos were saved as a playlist because search results on YouTube can change from day-to-day.

Videos with no audio English narration (n=7), those not related to colorectal cancer screening (n=17), duplicate videos (n=16), image only videos without voice, or audio only without images (n=12), and videos of less than 30 seconds (n=10) were not included in the study. Videos consisting of multiple parts were regarded as a single video (n=1). As a result of the search, 137 videos were included in the study.

The total number of views, the length, time elapsed after uploading and the numbers of likes, dislikes and comments were recorded for each video. The engagement of viewers with the video was calculated in terms of the number of views per day (this was determined by dividing the number of total views by the number of days after uploading). A new playlist was created for the videos included in the study.

We did not apply to any medical ethics committee for the approval of this study, according to the Declaration of Helsinki of the World Medical Association, as no patient data or material were used and all the videos used for the study were available on a public social media website (YouTube).

Evaluation of the videos’ usefulness

All 138 videos were independently evaluated using the Quality Criteria for Consumer Health Information (DISCERN) scale and Global Quality Scale (GQS), by three physicians (OY, MYU, NI). The three physicians blindly evaluated the videos and when there was a lack of consensus, a final decision was made by the fourth physician (EE). Subsequently, two groups were created. The group classifications were: (1) useful information (group 1) - the information was correct and they were helpful for learning CRC screening; (2) misleading information (group 2) - these included incorrect information about CRC screening (videos with an element of useful information were also included in this group because they did not contain any basic information about CRC screening.)

Classification of the Video Characteristics

The videos were separated into the following five groups according to the source of the upload: source 1 - academic-based channels (university and educational research hospitals); source 2 - non-profit associations and physicians/physician groups/professional organizations; source 3 - for-profit companies, private hospital publications, medical advertisements; source 4 - public spotlight videos; source 5 - TV health programs.

The total number of views, lengths, time elapsed after uploading and the numbers of likes, dislikes, and comments were recorded for each video. The engagement of the viewer with the video was calculated in terms of the number of views per day (this was determined by dividing the number of total views by the number of days after uploading).

A five-point DISCERN scale (0-5 points) was used to evaluate the reliability of the videos [14,15], and for evaluating the quality, a five-point Global Quality Scale was used (GQS: 1 = poor quality; 5 = perfect quality) [16]. A 14-point scale was used to evaluate the comprehensiveness of the video content in terms of current literature information. The parameters included in the scale were the risk groups, appropriate time intervals, types, characteristics, advantages and disadvantages of screening tests, and screening termination time (a separate score was kept for each step covered: not mentioned 0 points; briefly mentioned 1 point; mentioned in detail 2 points) (Table 1). Both scales were commonly used in previous YouTube analysis studies pertaining to various disesases [17-19].

**Table 1:** Evaluation tools for reliability, global quality, and comprehensiveness of the YouTube videos on CRC screening CRC: colorectal cancer

Reliability (if yes, 1 point for each equation)
1. Are the explanations clear and comprehensible in the video?
2. Are useful references provided? (Citations from credible publications)
3. Is the information in the video balanced and unbiased?
4. Are there additional sources of information from which the viewer can benefit?
5. Does the video provide information that is controversial or unclear?
The Global quality scale
1. Very poor quality: poor streaming; lacks most information; not useful for the patients
2. Fairly poor quality: provides some information; limited benefit to patients
3. Medium quality: adequately discusses some vital information
4. Good quality: good streaming; covers the most relevant information; useful for patients
5. Excellent quality: perfect streaming, very useful for patients
Comprehensiveness (not mentioned 0 point, briefly mentioned 1 point, mentioned in detail 2 points )
1. Clearly specifies low-risk and high-risk patients.
2. States the most appropriate time interval for colorectal cancer screening.
3. Explains which screening test will be selected and often the test will be repeated.
4. Specifies when the screening test will be terminated.
5. Explains the advantages of screening tests.
6. States the disadvantages of screening tests that could cause complications.
7. Mentions the false negativity or biased positivity of screening tests.

Statistical analysis

Normality testing was conducted for total views; video length; duration of stay on YouTube; the numbers of likes, dislikes, and comments; reliability score; comprehensiveness score, and global quality score; to check the assumption of normality of subgroups according to useful and non-useful video types. The normality hypotheses used in the statistical analysis were as follows: H0 - the variable is distributed normally; H1 - the variable is not distributed normally.

Since the normality distribution was not met by any variables, the comparisons were performed by using the non-parametric Mann-Whitney test. In the statistical evaluation conducted according to the source of the video, the multiple non-parametric Kruskal-Wallis test was preferred because no variables met the normality distribution assumption. A value of p < 0.05 was accepted as statistically significant.

## Results

The comparison test in terms of the total views revealed that the medians of the number of views in useful and misleading video groups were equal. No statistically significant difference was found between the two video groups in terms of the video length. There was no statistically significant difference between the two groups in terms of the duration of stay on YouTube. The difference in the number of likes was identified as significant between the two groups (p=0.013). It was understood that the number of likes was significantly higher in the videos containing useful information than the videos with misleading information. The comparison tests conducted for reliability, comprehensiveness, and quality scores revealed that the medians of the useful video group for all three were significantly higher (Table 2).

**Table 2:** Analysis of video characteristics by usefulness *Statistically significant.

Characteristics		Misleading information, n=63 (46%)	Useful information, n=74 (54%)	p-Value
Total views		412 (8:45403)	437 (17:88304)	0.17
Video length (s)		2.33 (0.3:8.56)	3.5 (1:9.53)	0.053
Duration on YouTube (day)		1146 (67:4070)	989.5 (54:3555)	0.612
Likes		1 (0:81)	2.5 (0:535)	0.013*
Dislikes		0 (0:9)	0 (0:28)	0.081
Comments		0 (0:38)	0 (0:126)	0.52
Comment-free videos		9 (56.3%)	7 (43.8%)	-
Viewer engagement with video		0.3 (0:23.6)	0.45 (0:84.4)	0.088
Source of upload, n (%)	Academic-based channels (university and educational research hospitals)	6 (14.6%)	35 (85.4%)	<0.001*
	Non-profit associations and physicians/physician groups/professional organizations	29 (72.5%)	11 (27.5)	
	For-profit companies, private hospital publications, medical advertisements	13 (41.9%)	18 (58.1%)	
	Public spotlight videos	2 (33.3%)	4 (66.7)	
	TV health programs	13 (68.4%)	6 (31.6%)	
Only stool-based tests are explained in the video		6 (50%)	6 (50%)	0.915
Only imaging tests are explained in the video		17 (43.6%)	22 (56.4)	
Both imaging tests and stool-based tests are explained in the video		63 (46%)	74 (54%)	

**Figure 1:**
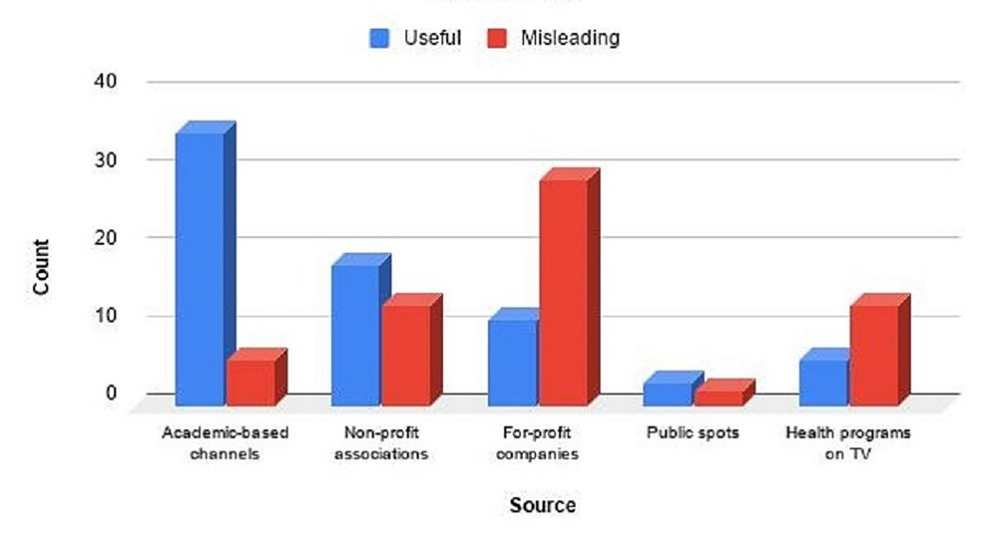
The useful/misleading ratios of videos according to the source

Since the p-value was below 0.05 in the chi-square test conducted according to the upload source, it was understood that the video source and information type variables were dependent variables. Hence, the useful/misleading ratio in videos from academic-based channels was prominently higher. In addition, the useful/misleading ratio was higher for the videos posted by non-profit associations and public spotlight videos. The videos of for-profit companies and TV health programs had a lower useful/misleading ratio (Figure 1).

There was a significant difference between the medians of the number of views according to the video source groups. In binary comparisons, it was seen that the public spotlight videos were watched significantly more than the non-profit association videos (p=0.01) (Table 3).

**Table 3:** Analysis of video characteristics by the source of uploads *Statistically significant.

Characteristics	Academic-based channels (university and educational research hospitals) 41 (29.9%)	Non-profit associations and physicians/physician groups/professional organizations 31 (22.6%)	For-profit companies, private hospital publications, medical advertisements 40 (29.2%)	Public spotlight videos 6 (4.4%)	Health programs on TV 19 (13.9%)	p-Value
Total views	361 (30:51700)	286 (8:8985)	479 (17:5361)	5395.5 (358:88304)	416 (22:44791)	0.023*
Reliability score	4 (2:5)	2 (0:3)	3 (1:5)	3 (0:3)	2 (0:3)	<0.001*
GQS score	4 (2:5)	2 (1:4)	3 (1:5)	3 (1:3)	2 (1:4)	<0.001*
Comprehensiveness score	9 (2:14)	3 (1:12)	5 (1:14)	5 (1:8)	4 (1:8)	<0.001*
Total view	361 (30:51700)	286 (8:8985)	479 (17:5361)	5395 (358:88304)	416 (22:44791)	0.023*
Video length (s)	2.4 (1.14:9.38)	2.56 (0.35:9.5)	3.4 (1.2:8.6)	2.2 (0.3:3.58)	4 (1:7.19)	0.169
Duration on YouTube (day)	1140 (54:3551)	772 (69:4070)	1453 (55:3555)	832.5 (580:1923)	991 (67:3015)	0.540
Likes	3 (0:535)	1 (0:19)	2 (0:71)	9.5 (2:79)	3 (0:81)	0.004*
Dislikes	0 (0:28)	0 (0:2)	0 (0:1)	1 (0:8)	0 (0:9)	0.143
Comments	0 (0:126)	0 (0:38)	0 (0:5)	0.5 (0:7)	0 (0:12)	0.082
Viewer engagement with video	0.3 (0:71.1)	0.4 (0:31.5)	0.3 (0:4.2)	9 (0.4:84.4)	0.3 (0.1:17.9)	0.026*
Only stool-based tests are explained in the video	1 (8.3%)	4 (33.3%)	4 (33.3%)	0 (0%)	3 (25%)	0.33
Only imaging tests are explained in the video	15 (38.5%)	9 (23.1%)	7 (17.9%)	1 (2.6%)	7 (17.9%)	
Both imaging tests and stool-based tests are explained in the video	25 (29.1%)	27 (31.4%)	20 (23.3%)	5 (5.8%)	9 (10.5%)	
Misleading information n (%)	6 (9.5%)	13 (20.6%)	29 (46%)	2 (3.2%)	13 (20.6%)	
Useful information n (%)	35 (47.3%)	11 (14.9%)	18 (24.3%)	4 (5.4%)	6 (8.1%)	<0.001*

A significant difference was observed between the video upload source groups for the reliability score. In binary comparisons, videos from non-profit associations were found to be less reliable than videos posted on academic-based channels (p=0.026). 

It was observed that there was a significant difference between the video upload source groups in terms of the comprehensiveness score. The binary comparisons indicated that the comprehensiveness scores of the videos by for-profit companies were lower than that of the videos by academic-based channels (p<0.001). Similarly, it has been observed that the TV health programs were less reliable than the videos in academic-based channels (p=0.001). In addition, videos produced by the non-profit associations were found to be significantly less comprehensive than those in academic-based channels (p=0.025).

It was observed that there was a significant difference between the video upload source groups in terms of the GQS quality score. Also, in the binary comparisons, it was detected that the quality scores of the videos based on TV health programs were lower than those posted by the academic-based channels (p=0.001 ). Similarly, it was found that there was a significant difference between the video source groups in terms of the number of likes. In the binary comparisons, it was established that non-profit organizations received a significantly lower number of likes than academic-based channels (p=0.023). In terms of video interaction, a significant difference was determined according to the video source. The binary comparison tests showed that the viewer interaction of the videos posted by the non-profit associations was significantly lower than that of the public spotlight videos (p=0.012). 

**Figure 2:**
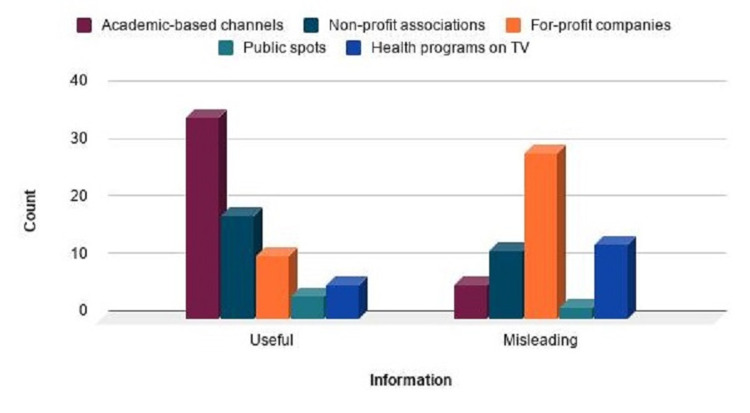
The sources of videos containing useful/misleading information

However, it was observed that the videos of not-for-profit associations received significantly lower interaction than the videos of for-profit companies (p=0.019). Additionally, it was also found that the academic-based videos had a significantly lower interaction than the public spotlight videos (p=0.044). It was detected by the chi-square test of independence that there was a significant relationship between groups (useful information group and misleading information group) and the video sources (Figure 2).

According to the results of the chi-square test, the rating of the videos of for-profit companies in the misleading information group was the highest. On the other hand, the rating of the public spotlight videos was the lowest. In the useful information group, the videos from academic-based channels had the highest rating, while the public spotlight videos had the lowest.

## Discussion

YouTube, which has been operating since 2005, is the most popular video-sharing platform among internet users today [20]. Although YouTube was originally a platform designed for entertainment purposes, in recent years, both healthcare providers and many members of the community have been using YouTube to provide information and learn about medical issues. 

Inadequate public awareness on CRC screening and cultural resistance to CRC screening tests are known screening challenges [21]. YouTube has been shown to be an actively used source by internet users for obtaining information about health issues [22]. It has been established that YouTube is an important platform, and one frequently used as an information source. There are few studies available in the literature about CRC screening related to YouTube [23,24]. These studies conclude that more comprehensive videos are needed. In addition, they emphasize that the number of views for videos on CRC screening is low and should be increased. Unlike the design for these studies, in the current study, we wanted to reveal the information quality of the first videos that are being encountered by users in an unfiltered search made for CRC screening, what the sources of these videos were, whether the most popular videos were adequate, and what was to be done to further and better inform the users.

At the end of the study, we made deductions under three main topics. In the useful information group, the highest number of videos were posted by academic-based sources. The length of these videos was long, the narrative had excessive use of medical terms, the visual content was poor and the memorable features were inadequate. For these reasons, although they contained the most useful and reliable information, we could see that the number of likes given by the viewers as well as the video interactions was very low. We are of the opinion that to be viewed more by the users, the academic-based videos should be shortened and visually enriched.

The for-profit companies uploaded the highest number of videos among the sources in the misleading information group. We consider that the reason for them to be included in the misleading information group is that for commercial reasons the videos are created to explain a single screening method, in order to highlight and market its own product. We conclude that the reason that the number of views is higher than those for the academic-based videos is due to the for-profit companies’ advertising agreements, as even YouTube users who are not searching with words associated with CRC screening see these videos on their pages.

The source of the video that the users viewed the most, following a standard search, was the public spotlight videos. Those videos had the highest number of views and the highest interaction rate. We established that the rate of videos that were viewed so much and had so many interactions was the least among the useful information videos. The content of these videos is poor because they explain only one to two CRC screening procedures. We think that public spotlight videos are the most-watched group due to their short length and the high amount of visual content. Previous studies have reported similar results [17-19].

In the present study, we ascertained to what extent videos belonging to which source were viewed. The public spotlight videos were the most viewed videos and it was concluded that their content should be increased. In addition, we detected that the academic-based videos contained comprehensive information, but even so were less viewed compared to videos from other sources. We believe that the artificial intelligence infrastructure of YouTube could be used to increase the number of views of those videos, and cooperation could be sought with the YouTube website management in this regard.

In addition, we think that it will make a difference if high-quality videos that have received referee approval, in terms of CRC screening, are included in the suggestion videos section spontaneously, for YouTube users of the screening age using algorithms.

Study limitations

First of all, the data included in the study were extracted at a certain time and as is known, view, like, dislike, and comments change every second. Second, only English language videos were evaluated, making generalization of the results difficult. Finally, we used the term "colorectal cancer" (CRC), which may not be exactly understood by layperson. However, we had to use it because the primary objective of the study was the evaluation of the videos about CRC. Nevertheless, we believe that our results will contribute to what is known on the issue in the literature.

## Conclusions

We think that the CRC screening rate could increase by collaborating with YouTube (reaching more people by means of algorithms), enriching the public spotlight videos with useful information, keeping academic videos short and more viewable. In addition, healthcare professionals should be encouraged to upload accurate and useful videos to YouTube in order to direct those who want to inquire about screening to professionals. Furthermore, YouTube authorities should develop new strict policies and strategies to inspect and audit particularly health-related videos before they can be published.
